# Communicating Bad News: Insights for the Design of Consumer Health Technologies

**DOI:** 10.2196/humanfactors.8885

**Published:** 2019-05-17

**Authors:** Eun Kyoung Choe, Marisa E Duarte, Hyewon Suh, Wanda Pratt, Julie A Kientz

**Affiliations:** 1 College of Information Studies University of Maryland College Park, MD United States; 2 School of Social Transformation Arizona State University Tempe, AZ United States; 3 Human Centered Design and Engineering University of Washington Seattle, WA United States; 4 Information School University of Washington Seattle, WA United States

**Keywords:** mobile health, eHealth, mHealth, patient-centered care, health communication, empathy

## Abstract

**Background:**

As people increasingly receive personal health information through technology, there is increased importance for this information to be communicated with empathy and consideration for the patient’s experience of consuming it. Although technology enables people to have more frequent and faster access to their health information, it could also cause unnecessary anxiety, distress, or confusion because of the sensitive and complex nature of the information and its potential to provide information that could be considered bad news.

**Objective:**

The aim of this study was to uncover insights for the design of health information technologies that potentially communicate bad news about health such as the result of a diagnosis, increased risk for a chronic or terminal disease, or overall declining health.

**Methods:**

On the basis of a review of established guidelines for clinicians on communicating bad news, we developed an interview guide and conducted interviews with patients, patients’ family members, and clinicians on their experience of delivering and receiving the diagnosis of a serious disease. We then analyzed the data using a thematic analysis to identify overall themes from a perspective of identifying ways to translate these strategies to technology design.

**Results:**

We describe qualitative results combining an analysis of the clinical guidelines for sharing bad health news with patients and interviews on clinicians’ specific strategies to communicate bad news and the emotional and informational support that patients and their family members seek. Specific strategies clinicians use included preparing for the patients’ visit, anticipating patients’ feelings, building a partnership of trust with patients, acknowledging patients’ physical and emotional discomfort, setting up a scene where patients can process the information, helping patients build resilience and giving hope, matching the level of information to the patients’ level of understanding, communicating face-to-face, if possible, and using nonverbal means. Patient and family member experiences included internal turmoil and emotional distress when receiving bad news and emotional and informational support that patients and family members seek.

**Conclusions:**

The results from this study identify specific strategies for health information technologies to better promote empathic communication when they communicate concerning health news. We distill the findings from our study into design hypotheses for ways technologies may be able to help people better cope with the possibility of receiving bad health news, including tailoring the delivery of information to the patients’ individual preferences, supporting interfaces for sharing patients’ context, mitigating emotional stress from self-monitoring data, and identifying clear, actionable steps patients can take next.

## Introduction

### Motivation

The proliferation of health technologies—such as fitness trackers, self-monitoring tools, and personal health records (PHRs)—enables people to be aware of their own health information more than ever before. The information patients may gather about their health from such technologies includes a casual notice of weight gain or loss, changes in cholesterol or blood glucose levels, signs of developmental delays, or an increased risk of a serious disease such as diabetes or Alzheimer’s. Having access to personal health information via various technology channels can help people manage chronic conditions, encourage healthy habits, or bring awareness to problems they might not have previously recognized. Although people have frequent and fast access to their health data, the tools have the possibility of communicating bad health news without consideration for the patients’ emotional condition to which a skilled clinician can be responsive. For example, with PHRs, patients can check their laboratory results on the Web without the presence of clinicians [1]. In the absence of the human element, such as the informational or emotional support that can take place during communicating health news in-person by a skilled physician, people could have difficulty assimilating information and making informed decisions about treatment options, lifestyle changes, and medications that could create undue emotional burden on patients. These situations could be avoided if health technologies are designed with *empathy*, which is known to positively influence patient health outcomes such as patient satisfaction and adherence to treatment [2].

In this paper, we argue that health information systems that potentially communicate bad health news need to deliver the news while considering the emotional needs for patients and that such needs have been largely unfulfilled in the design of current health information systems [3,4]. By investigating *how clinicians communicate bad news* about health, we can learn and apply strategies for designing health information technologies that are more empathic and can reduce the patients’ emotional burden. In this paper, we first review established guidelines and protocols for communicating bad news that are designed to train clinicians to improve their communication skills. We then discuss the semi structured interviews we conducted with clinicians and patients to understand their respective experiences of delivering and receiving a diagnosis for severe or chronic conditions—such as cancer, Parkinson’s disease, or diabetes. We identify and characterize the issues around health technologies that potentially cause patients anxiety, distress, and frustration and identify patients’ and their caregivers’ emotional and informational needs at the time of receiving “bad news.” We discuss design hypotheses and example designs that leverage the strategies suggested by participants and guidelines from patient-clinician communication literature [5-10].

### Strategies and Technologies for Communicating Health News

#### Clinical Guidelines for Communicating Bad Health News

Although communicating bad news is an important part of medical care, both clinicians and patients find it difficult. Clinicians have legal and ethical obligations to provide patients with as much information as they want [11] even if they suspect that it will have a negative impact on patients. A majority of patients desire to be told the truth about the diagnosis of a serious disease (eg, cancer) and even a grave prognosis [12]. As clinicians find it challenging to be honest with their patient and not destroy the patient’s hope at the same time, many guidelines recommend how to communicate bad news.

Guidelines for communicating bad news are developed on the basis of reviews of other literature [7,13] and clinical opinions [14,15]. Although rare, a few studies account for patients’ opinions [7,16]. Some guidelines are geared toward specific medical situations—such as communicating to cancer patients [14] or parents of a child with additional needs [17,18]. However, in general, communication skills are not disease-specific knowledge, and thus established guidelines can be applicable to a wide variety of situations where clinicians across specialties communicate with patients. Communication guidelines comprise ways to set context, listen to patients, acknowledge their emotions, and share medical information. It has been found to be useful in all medical interviews—especially in palliative care and psychotherapeutic dialogue—as the “breaking of bad news is universal to medicine” [8].

Communication guidelines and models assume that communication skills can be taught and acquired. Since the 1990s, North American medical schools began to teach communication skills. According to a 1999 survey in which 89 of the 144 medical schools participated, 85% reported that they teach communication skills [19]. Of the schools that used a structured model in teaching communication skills (32%), 2 models they commonly used were the SEGUE (short for *S* et the stage, *E* licit information, *G* ive information, *U* nderstand the patient’s perspective, *E* nd the encounter) framework for Teaching and Assessing Communication Skills [20] and the Calgary-Cambridge observation guide [21]. As these models were general communication models, we looked for communication models specific to breaking bad news that are widely used in the medical community—the SPIKES model [14] and Consensus Guidelines [7]—and used them for framing our interview guides and analysis. The SPIKES model is useful for its simplicity, and the Consensus Guidelines are useful because of their comprehensiveness. The SPIKES 6-Step protocol emphasizes the sequence of communicative acts occurring alongside a process of emotional acknowledgment and repositioning. It comprises the following steps: (S)—Setting up the interview; (P)—assessing the patient’s Perception; (I)—obtaining the patient’s Invitation; (K)—giving Knowledge and information to the patient; (E)—addressing the patient’s Emotions with empathic responses; and (S)—using Strategies and Summary [14]. Detailed strategies are provided within each step—strategies for “setting up,” for example, include arranging for privacy, involving significant others, and managing time constraints and interruptions; strategies for “obtaining the patient’s invitation” refer to the process of determining how much information a patient wants to know and when they want to hear it. This guideline is based on the grounds that everyone has different information needs and that clinicians should ask questions (eg, “How would you like me to give you the information about the test results?”) to gauge how much information a patient wants to know. The SPIKES protocol has been incorporated into a variety of training programs for clinicians and medical students across many disciplines. It has been evaluated by patients according to their rating of the procedure, perception, and satisfaction [22].

One caveat with the SPIKES protocol is that it is developed on the basis of communication techniques rather than empirical evidence. The consensus guidelines [7] on the other hand take a different approach of reflecting the clinicians’ and patients’ opinions during the process of developing the model. After a critical review of the medical literature on how to communicate bad news, the authors developed a draft of guidelines and then presented them to a consensus panel of medical professionals (n=28) and patients diagnosed with cancer (n=100) for their feedback. The consensus guidelines are a list of attributes rather than a sequence of communicative acts. They offer distinct guidelines such as being sensitive to patients’ cultural, religious, or social background, employing a trained health interpreter if necessary, encouraging the patient to express his or her feelings and documenting what the patient has been told. Textbox 1 summarizes the consensus guidelines [7] for communicating bad news.

**Consensus guidelines.**

Summary of recommendations for communicating bad news

- One person only should be responsible for breaking bad news
- The patient has a legal and moral right to information
- Primary responsibility is to the individual patient
- Give accurate and reliable information
- Ask people how much they want to know
- Prepare the patient for the possibility of bad news as early as possible
- Avoid giving the results of each test individually, if several tests are being performed
- Tell the patient his or her diagnosis as soon as it is certain
- Ensure privacy and make the patient feel comfortable
- Ideally, family and significant others should be present
- If possible, arrange for another health professional to be present
- Inform the patient’s general practitioner and other medical advisers of the level of development of patient's understanding
- Use eye contact and body language to convey warmth, sympathy, encouragement, or reassurance to the patient
- Employ a trained health interpreter if language differences exist
- Be sensitive to the person’s culture, race, religious beliefs, and social background
- Acknowledge your own shortcomings and emotional difficulties in breaking bad news

The review of the guidelines reveals a considerable overlap between SPIKES and the consensus guidelines such as ensuring privacy, assessing the patient’s understanding of the situation, and providing an honest diagnosis using simple language. In a clinician’s attempt to understand what it is like to be a patient, active listening and expression of feelings are the hallmarks of empathy during clinician-patient communication.

In our research, we used a review of the clinical guidelines to help frame our interview guides and as a starting point for our thematic analysis.

#### Patients’ Preferences for Communicating Health News

Several studies have investigated patients’ preferences for receiving health news, specifically in the context of receiving cancer diagnosis during the in-person communication [23-25]. For example, Parker et al conducted a survey to understand the characteristics of communication that different types of cancer patients would prefer such as what and how much information to receive, what setting and context they want to be in, and whether to receive emotional support during the communication [25]. Although these studies generate useful suggestions for improving in-person communication (eg, “Establishing a basis for breaking bad news” [23]), our goal is to identify insights for technology design. In this regard, Choudhry et al provide intriguing findings from their study on patients’ preference for receiving skin biopsy results (which might contain a malignant diagnosis)—majority of patients (67.1%) preferred to receive the news via a telephone over other methods such as face-to-face communication (19.5%) or patient portal (5.1%) [26]. Top 2 contributing factors were (1) wanting to receive the results in the most rapid manner and (2) wanting to have an opportunity to ask questions when needed. In designing technologies for communicating bad news, we believe that these 2 aspects are important design considerations that need to be supported.

#### Self-Monitoring Tools for Health

Self-monitoring tools for health—such as blood glucose meters, electronic scales for body weight and body fat percentage, devices for sleep behavior patterns, and journaling tools for food—have proliferated in recent years. These self-monitoring tools often help people increase awareness of their behavior, identify patterns of behaviors, manage chronic conditions, or observe the effects of treatment. Self-monitoring tools could also improve the chances of early detection of a disease, which could also increase the chances of successfully treating it [27]. The real benefit of self-monitoring comes from using it on a regular basis long enough to identify trends. However, tracking data over time could cause anxiety when the data do not meet the observer’s expectations, when the data show that the user is out of the normal range or if the user misinterprets or makes incorrect inferences from data [28]. Recent research has explored the phenomenon of people bringing self-monitoring data to their provider, but that presents a number of challenges [29,30] such as increased burden for both patients and providers, privacy concerns, and perceived disruption of a provider’s primary care duties.

#### Personal Health Records and Electronic Medical Records

PHRs allow individuals to take an active role in managing their health and keeping their health information up-to-date [31]. Integrated PHRs—often referred to as patient portals or tethered PHRs—include a subset of health data from electronic medical records (EMRs) and provide more diverse features than an independent PHR. For example, they allow patients to access their laboratory test results, schedule appointments, or request prescription refills. Currently, the types of information that should be shared and how the information should be released have been the subject of heated debate [32]. Some clinicians are not enthusiastic about patients’ direct access to their health information—such as laboratory test results and doctors’ notes [33], despite their legal and ethical obligation to provide information if a patient asks for it. Clinicians worry as health information shared on the Web could potentially convey bad news to a patient, and thus patients run the risk of anxiety either with too little information (because of limitations of electronic media) or overwhelming information (in case of abnormal test results that may be difficult for a nonexpert to interpret). However, patients desire to have direct access to health information, including normal and abnormal test results, in less time than current norms [34]. Patient advocates argue that patients’ direct access is a quick and efficient way of sharing information and might improve patient understanding and involvement in care [35,36]. Although we argue that health information systems should provide patients with direct and timely access to their own health information, this study offers design considerations for interfaces to minimize some of the negative consequences of such access on the patients’ side.

#### Affective Computing

Affective computing approaches consider empathy as a physiological or behavioral measure and interpret those measures as emotions [37-39]. Studies in the affective computing literature often describe agent-based systems with animated humanoid software that emulates empathy through verbal and nonverbal modalities in various contexts. Agent-based systems are designed to alleviate a computer user’s frustration [40], deliver discharge information in place of clinicians [41], or reduce stress levels of job interviewees [42]. Studies indicate that computers with such abilities can draw positive user reactions and increase people’s desire to continue using the system. However, other studies show that agent-based systems are not yet sophisticated enough to replicate the subtlety and complexity of human empathy [43]. Boehner et al [44] assert that design should shift “from helping computers to better understand human emotion to helping people to understand and experience their own emotions.” Although affective computing approaches concentrate on designing relational agents that emulate empathy, we aim to uncover opportunities for health technologies that support an empathic human-human relationship.

#### Emotional Support Through Health Technologies

We note that evaluation measures for health information systems are heavily weighted toward traditional usability (eg, screen layout) and efficiency (eg, learning ability, cost-effectiveness, task completion time, and error rate) aspects [45,46], and they often neglect how the system supports patients’ emotional and mental states [47], though a recent study by Suh et al included emotional burden within their User Burden Scale for computing systems [48], and Kientz et al described considering emotional impact in the design of persuasive technologies [49]. For information and communication technologies studied in hospital settings, designers aim to improve clinicians’ work efficiency or data entry [4,50], but they often neglect to support the emotional needs of patients. One exception is the study by Toscos et al, which highlights the importance of considering diverse emotional needs when designing health-monitoring technologies for teens with diabetes and their parents [51]. Technology has great potential to provide space for patients’ emotional support. Researchers describe empathy as common in online patient support groups where patients seek both emotional and informational support [52,53]. Others reveal various characteristics of empathy presented in online discussion boards [54], or they have designed virtual agents to help convey empathy toward patients in care settings [41]. We can learn from these existing tools in the design of new health technologies.

### Aim of the Paper

The primary goal of this research is to understand the design requirements for and investigate specific strategies for improving consumer-facing health technologies to communicate health news to patients in a way that is more empathetic and in line with best practices from clinical work in this space. The development of these requirements and strategies requires an empirical understanding of experiences of patients, clinicians, and patient family members.

## Methods

### Interviews With Clinicians and Patients

To understand the design space of using technology to communicate bad health news, it was critical for us to have firsthand dialogue with those who are involved in the process of delivering and receiving news about one’s health. We thus conducted semistructured, open-ended interviews with clinicians, patients with chronic conditions, and patients’ family members to better understand their experience and enable us to translate the findings from the medical guidelines into more practical considerations for our own work. Researchers from the medical field have conducted interview studies involving patients and patients’ family members; however, these studies were aimed at developing guidelines for the clinicians [7,14], whereas our interview study is aimed at identifying opportunities for health information technology design. Moreover, clinicians’ views (eg, feelings, thoughts, and behaviors clinicians have when delivering bad news) were studied mostly using structured surveys [14,55,56]. Therefore, it was important to include interviews with the 3 key stakeholders—patients, patients’ family members, and clinicians. We chose to do a retrospective perception study rather than a study based on direct observation of clinician-patient communication as we considered asking patients or family members how they felt immediately after receiving bad news to be unethical and impractical. Although studies focusing on the communication of bad news are typically based on retrospective recall [57], we acknowledge that this approach has limitations—such as recall bias.

#### Recruitment

We recruited participants through word-of-mouth sampling and Craigslist postings in the United States. We interviewed a total of 23 participants—8 clinicians, 1 medical student, 1 social worker, 9 patients, and 4 patients’ family members (see Table 1). Throughout the paper, we use the following naming scheme: “Cx” for clinicians, “Px” for patients, and “Fx” for family members. We offered a US $20 gift card to interviewees in appreciation for their participation. During screening, we sought clinicians or social workers who regularly conducted in-person medical diagnoses, prognoses, or consultations with patients. The social worker in this study had 12 years of experience in delivering the news of positive HIV tests to clients, and thus added a broader perspective than those trained as MDs (Doctor of Medicine) or nurses. We also sought patients who had been diagnosed with severe or chronic conditions. Although we did not formally define “severe or chronic conditions” in the recruitment posting, we listed cancer, Parkinson’s disease, and diabetes as examples of these conditions, and we let patients self-identify what they considered as severe or chronic conditions. As there is limited literature reflecting the perspectives among clinicians, patients, and family members, we chose to include all 3 participant groups in this study. In addition, as empathic communication is universal across different conditions in health care, we expected that a diverse sample would give us insights into the variety of ways it manifests.

**Table 1:** Demographic details of participants.

ID^a^	Group	Age	Gender	Area of expertise or type of condition	Years, months, or weeks of experience
C1	Doctor	62	M^b^	Oncology	25 years
C2	Doctor	59	M	Women’s health	25 years
C3	Med. student	32	M	Internal medicine	4 years
C4	Nurse	34	F^c^	Intensive care unit	3 years
C5	Doctor	45	M	Family medicine; psychiatry	19 years
C6	Doctor	39	M	Pediatric cardiology	11 years
C7	Doctor	—^d^	M	Internal medicine	14 years
C8	Nurse	—	F	Family health nurse	13 years
C9	Doctor	34	F	Internal medicine	10 years
C10	Social worker	45	F	Delivering HIV test results	12 years
P1	Patient	50	F	Parkinson’s disease; breast cancer	17 years
P2	Patient	39	F	Diabetes; gastroparesis	27 years
P3	Patient	56	F	Follicular lymphoma—stage 4; diabetes	5 years
P4	Patient	57	F	Parkinson’s disease; breast cancer; knee replacement	12 years
P5	Patient and Family	45	M	Himself—Thyroid cancer, Wife—ovarian cancer	Patient: 1 year; Caregiver: 2 years
P6	Patient	21	F	Heart disease	2 years
P7	Patient	34	F	Muscle disease (peripheral myopathy); Crohn’s disease	1 year
P8	Patient	60	F	Uterine cancer; breast cancer	12 years (uterine cancer); 8 years (breast cancer)
P9	Patient	22	M	Bone cancer (Ewing’s Sarcoma)—stage 4	1 year
F1	Family	45	F	Partner of P3	5 years
F2	Family	43	F	Sister was diagnosed with diabetes	2 weeks
F3	Family	43	F	Mother was diagnosed with liver cancer (stage 4) and passed away	1 month as a caregiver
F4	Family	35	F	Son was diagnosed with type 1 diabetes when he was 12 months old	3 years as a caregiver

^a^Naming scheme: “Cx” for clinicians, “Px” for patients, and “Fx” for family members.

^b^M: identifies male.

^c^F: identifies female.

^d^Age of provider was not given.

#### Interview Protocol

During the interview, clinician questions addressed the following: (1) perceptions of bad news, (2) diagnosis process, (3) strategies to deliver bad news, (4) common patient reactions and their coping strategies, and (5) perspectives on empathic care. We modified the interview questions for patient and family participants and asked the following: (1) the moment they heard the bad news and how it was communicated, (2) thoughts and reactions in receiving the news, (3) ways to manage and reduce distress, (4) the role of family members, and (5) memorable encounters with clinicians, either good or bad. All interviewees were encouraged to walk us through a specific case. Of the 23 interviews, 8 were conducted in person and the rest via phone. Interviews lasted from 30 min up to 2 hours.

### Analysis

We audio recorded and transcribed all interviews to aid with analysis. We employed cross-case analysis of the transcripts using a thematic analysis approach [58]. During the interpretation phase, 2 researchers independently read through the transcripts and identified themes. The researchers then vetted, defined, and merged the themes into 1 code set. Using the preliminary code set, the 2 researchers independently coded the transcripts using Text Analysis Markup System [59]. Overall, 2 researchers exchanged the coded transcripts and reviewed the other’s codes. The research team met regularly to discuss new themes and refine preexisting categories in the code set, thereby iterating on the codebook. The final, high-level categories of the analysis were characteristics of bad health news, strategies that clinicians use to express empathy (understanding and communicating), patients’ experiences and reactions in receiving bad news, patients’ perspectives on poor communications of bad health news, and information and emotional support for patients and family members. We then used the analysis of the interviews combined with our review of clinical guidelines to develop our design guidelines for interactive technologies.

## Results

### Characteristics of Bad News

Bad news in the context of medical situations is defined as “any information which adversely and seriously affects an individual’s view of his or her future” [60]. Bad news is in the “eye of the beholder,” such that different people receive it differently depending on their life experience, personality, spiritual belief, philosophical standpoint, perceived social support, and emotional hardiness [57]. Clinician participants defined bad news as patients having a very serious illness, disease with poor prognosis [C6], or problem associated with the illness (eg *,* suddenly becoming blind from diabetes) [C5]. How people perceive bad news is context dependent. For example, bad news could be perceived as more tragic in young patients [C5], such an unexpected health condition affecting an infant, as opposed to the same condition affecting an older adult who already experienced related conditions. Moreover, not all bad news is perceived as tragic; if a disease is treatable or easy to manage, bad news could be heard as good news. P5 described as follows:

I had only thyroid cancer, not the lymphoma, which is very good news.

P5

Some participants [P4 and F4] even felt a *sense of relief* when they finally got a concrete diagnosis of a disease. On the other hand, a clinician being uncertain of what the patient has evokes anger and frustration on the patient’s side. For example, P8 had a muscle disease, but her doctor did not know what type of muscle disease she had, even after many laboratory tests. This situation was frustrating for P8 as she did not know how to tell other people what medical condition she had or with which support group she could connect. However, a clinician participant had a different view. According to C9, not having a concrete diagnosis could turn out to be good news after all:

There are a lot of times when the diagnosis isn’t sure, and that usually has a better prognosis. If I had a weird symptom, I’d prefer not knowing what it is, because chances are, it’s not that bad in terms of statistics. It’s counterintuitive, I agree. It’s not the way we think, usually. But that’s only because I know we’ve done the right tests...ruled out the bad things. Chances are, it’s getting better.

C9

As such, how people perceive bad news is different for every person. Clinicians describe “bad news” in the objective sense on the basis of the severity and prognosis of the disease. On the other hand, patients and family members respond to bad news rather subjectively depending on many factors—such as past experience, expectation, personality, and religion.

### Clinical Empathy and Empathic Communication

#### Definition of Empathy and Characteristics of Empathic Communication

The clinicians’ empathic communication skill was particularly important in delivering bad news for both clinicians themselves (eg, a decreased risk of litigation) and patients (eg, lessening the distress). Clinicians in this study described empathy, in many ways, such as how C3 described it:

Understanding how you would feel if you were in the same situation as somebody that is going through an illness.

C3

C6 described it as:

Humanizing the diagnosis and the procedure

C6

C4 described it as:

Treating people like human beings rather than treating people like an illness.

C4

Finally, C3 described it as the following:

A clinicians’ empathic communication skill is “more like an art than a science".

C3

A clinician’s empathic communication skill requires the ability to create a connection with people that is beyond just clinical information. When we asked clinician participants if being empathic to patients can be learned, many agreed that empathic communication is indeed a learnable skill.

Experienced clinicians are well aware of the intrinsic value of empathic communication—the recursive process of *understanding* and *communicating* with patients—which is different from the step-by-step process that the SPIKES protocol suggests [14]. Empathy is hardly ever communicated without the clinician’s understanding and acknowledgment of the patient’s context. For example, the clinician might need to know the patient’s feelings, level of understanding of the disease and options, work situation, and home life. Furthermore, the clinician’s understanding of a patient’s situation and emotional state means little unless the clinician is able to skillfully communicate that understanding. Understanding and communicating happen simultaneously as clinicians consciously and continuously reassess the patient’s situation. Confirming the guidelines, clinician participants said they modify their method of delivering unexpected news on the basis of the patient’s feedback and life story. However, C8 stated that modifying the method of delivery is often hard to achieve in the intensive care unit where patients rely on a ventilator and other supporting devices and often cannot communicate directly with clinicians.

#### Strategies to Understand Patients’ Context

We identified that empathic clinicians make an effort to understand patients’ context *before* and *during* the patients’ visit.

##### Preparing for the Patients’ Visit

Before meeting with patients, clinician participants reported a need to remind themselves of the patient’s situation by checking the patient’s chart, reviewing information, and looking for certain characteristics (eg, the disease, laboratory results, records of previous procedures, or other key events). Patients’ occupation or cultural background was additional information that helped clinician participants adjust their way of speaking to accommodate patients’ medical understanding.

##### Anticipating Patients’ Feelings Through Careful Observation

In preparing to deliver unexpected and life-changing news to patients, clinician participants reported not only anticipating the patients’ level of medical understanding but also acknowledging patients’ feelings. The 5 stages of grief model (denial, anger, bargaining, depression, and acceptance) by Kubler-Ross [61] was often referenced during the interviews with clinician participants when they explained the importance of knowing where patients are in their feelings. Knowing where patients are in this model by looking into patients’ eyes helped clinician participants assess patients’ feelings and gauge what information to reveal when. C5 and C6 described as follows:

You have to watch them, and watch their faces. You have to try at least to read and get a feel for where they are with the conversation, is the first step.

C5

Another clinician said the following:

So I try to tell them as much as I can...but I gauge it on the family and the parents, and I try to watch them and look at how much I'm giving them and how they're reacting because it can be...it's very overwhelming.

C6

The clinicians we interviewed stated that knowing where patients are at emotionally helps them work around the state of shock and anxiety that often prevents patients from fully absorbing critical information. When clinicians perceive that patients are emotionally charged, clinicians might step back and wait for a better time to reveal certain information, invite patients to call with questions, or suggest that peers and family be present to help ask questions or make sense of the information. In this sense, the clinicians’ ability to empathize with patients is what helps the clinicians aid patients in assimilating troubling information.

After achieving an *understanding* of patients’ context through pre visit preparation and anticipation of patient’s emotional state through careful observation during a consultation, clinicians should be able to skillfully communicate that understanding. In what follows, we describe the strategies clinicians use to *communicate* with patients, which are the other important part of empathic dialogue.

#### Strategies to Communicate With Patients

Communicating empathy refers to clinicians’ acknowledgment of patients’ feelings. Clinician participants described several communication techniques they use to convey empathy while presenting information directly and simply, which aligned well with the clinical guidelines we analyzed.

##### Building a Partnership of Trust With the Patient

Clinician participants reported they commonly use their opening statement to reinforce a partnership. Clinicians want patients to trust in the quality of their care. Trust between patient and clinician alleviates patient fear, which could smooth the decision-making process that must occur around every new piece of clinical health information. To build a partnership of trust with patients, several clinician participants mentioned using language that reinforces an “us” relationship rather than a clinician versus patient hierarchy. The following examples show how clinicians reinforce a partnership, as stated by C1:

All of us are advocates for the baby and you.

C1

And as reported by C2:

I'm glad you came. Let's look at that report. Let's look at it together.

C2

And finally, by C5:

I'm gonna have to tell you something that's difficult and I'll give you all the details so that you understand it. I want you to know that we'll work with you to make sure you really fully understand it.

C5

##### Acknowledging Physical and Emotional Discomfort

Another way to communicate empathy is to address the patients’ feelings directly. Clinician participants reported using comments such as the following provided by C2:

It must be hard to encounter something that may seem so serious...I am sorry you are in pain. I hope we can work to make you to feel more comfortable.

C2

And the following by C5:

I could imagine how frightening this is to you.

C5

However, 1 clinician participant expressed the difficulty of having to maintain a certain distance from patients but wanting to empathize with their feelings at the same time:

It can be very tough if you become emotionally involved with the patient. For me personally, I try not to get completely tied in with them, but at the same time, I don’t want to not be saddened by telling a parent that their child is going to or has died. If I ever get to a point when I have a conversation with a family delivering them news of a prognosis and it doesn’t affect me, that would worry me that I’m too disconnected.

C6

Others observed how even in situations where a clinician could not save a patient’s life, the clinician’s empathic acknowledgment of the difficult situation made family members feel that they were being treated as human beings.

As I remember, hearing her deliver those news in such a loving, caring, compassionate way...“I care for you, I’m saying something that is very hard, I will be with you, there’s nothing we can do really to avoid the ultimate result, but we will work together to make it the best for you that we can.” And she was true to her words. She was there all the time. And we could thank her for having been there with us for 2 years.

P5

Although P5’s wife passed away, P5 was grateful to the wife’s clinician for the empathic approach to providing patient care. As such, a clinician’s acknowledgment of patients’ and family members’ emotional feelings helps them deal with bad news and go through tough times.

##### Setting up a Scene Where Patients Can Process Information

Creating a space for empathic dialogue between clinicians and patients requires that patients be in a comfortable and private environment where they can process the information being conveyed. A clinician participant [C9] described how she prepares to communicate bad news to an inpatient. After she makes sure that the patient is in a private environment, she does the following:

There’s usually a short social phase, where you talk to the person about how they stay at the hospital...you find something to make everyone feels at ease, you make sure whether they are sitting comfortably...you sort of unconsciously check that there’s tissue somewhere in the room if it’s really bad that you are gonna be announcing. Um...there’s usually tissue in your pocket or something...you know, that might be an issue...having to get up to go find tissue is not as nice afterwards. So as much as you can plan before, but that’s just a small thing that you just learn with time. That’s not in the textbook.

C9

According to our participants, the actual diagnosis is the most important piece of information for patients. The same information could be delivered in various ways—from people in different positions using different means of communication, and those ways affect the conveyed empathy. One patient participant received an unexpected phone call from a nurse saying the following:

Hi, we just wanted to let you know that the biopsy came back and it is a cancer.

P4

Others were informed by an experienced clinician who carefully revealed the diagnosis along with descriptions of the condition. The clinician then opened a dialogue wherein the patient and clinician could discuss treatment options, prognoses with and without treatment as well as what the patient could expect to go through with surgical procedures, side effects, expectations for healing, and lifestyle changes. The clinician’s goal in creating a time and space for empathic dialogue is to ensure that a patient fully understands his or her condition to make informed decisions without becoming overwhelmed in the clinical details.

##### Building Resilience and Giving Hope

The experienced clinicians described the importance of developing the patient’s emotional strength as that is what makes patients endure painful or chronic conditions; a clinician stated the following:

...the will to fight

C1

Even though it is discomforting for clinicians to tell patients the following:

This is what you will die from

P5

All clinicians we interviewed stated the importance of being honest, clear, and straightforward when delivering diagnoses. What is more important yet difficult is to obtain the balance between being honest about a poor prognosis and giving hope at the same time. Giving hope is different from giving false hope, which several participants also referred to as “sugarcoating.” Sugarcoating is telling patients glossy stories and assuring them that everything will be fine when in fact the patient is in failing health. All clinician participants asserted that sugarcoating is harmful for patients, and it only protects clinicians who want to avoid dealing with the patient’s emotions. However, giving hope helps in a situation when patients have to develop both the physical and emotional strength and resilience to endure a difficult situation. The gynecologist we interviewed told a story in which he encouraged a cancer survivor to consider undergoing a high-risk surgical procedure that would dramatically alter her physically but would also extend her life. He stated the following:

I looked at this woman—tremendous will, tremendous spirit—I brought her back and said, “There’s something that can be done. It’s a very radical surgery, and not many survive it. But those who do, they do well. So I need you to consider this. It means having an operation to remove ovary, bladder, vagina...all of them will be cleaned out...you will be sick, you will be in hospital for many days...but you may live. I think that you are tough and you can make yourself come through this. Are you up to this challenge? I think you can do it. I think you have it in you.” In turn, this patient needed one more chance of hope. So she got operated, and she survived.

C2

The quote above illustrates not only the clinician’s confidence in the patient’s capacity to endure a radical procedure on the basis of his previous knowledge of the patient’s life story and medical history but also the level of trust in the clinician-patient relationship that allowed him to speak with honesty and candor about the surgical outcome. Clinicians’ knowledge about their patient’s life story and their ability to communicate with such candor and trust helps patients build resilience and hope, which are indicative of empathic dialogue.

##### Matching the Level of Information to the Patients’ Level of Understanding

The experienced clinicians we interviewed present complex information in plain language to do the following:

...make the person in charge of their situation.

C2

In addition, to give patients the following:

...good information so they can make good judgments about their lives.

C1

When explaining data, clinicians break difficult concepts into down-to-earth terms and use visual aids such as drawings, graphs, pictures, and x-rays. Another strategy the clinicians used was to tailor their language to the patient’s life experience. For example, 1 clinician described speaking in probabilistic terms with patients who, as engineers, appreciated the mathematical explanation.

##### Communicating Face-to-Face, if Possible, and Using Nonverbal Means

All clinician participants explained the importance of face-to-face communication and being mindful of nonverbal communication during consultation. They preferred face-to-face communication with patients in a quiet, private space where they could maintain eye contact and, if needed, sit beside patients to look at data together. Some clinicians said they do not allow sensitive information to be delivered over the phone or by staff members who do not know how to communicate empathically. C10, who worked at an HIV clinic, also mentioned that it was the clinic’s policy to never give news over the phone regardless of the test outcome. Observing the patient’s (or client’s) body language and facial expression allows clinicians to tailor the way they give a message to individual patients.

However, face-to-face communication is not always possible if a time-critical test result comes back outside office hours or between the scheduled appointments. In addition, time constraints often limit the face time during appointments. Indeed, a few of our patient participants received their diagnosis over the phone, and some of them were grateful for their clinician’s attempt to reach them as soon as possible when the information was urgent. A patient stated the following:

What I still appreciate about my doctor at that point is that she called me. She actually called me while I was at work. And she called and asked if I was alone. “(Name of P8), I have some news for you that the test showed that you have a uterine cancer.” I appreciated her honesty, and that she called me. (...) She asked me if I was alone, and there was a part or piece in that she knew me well because I would not have wanted to get that information while other people were in the office and I wanted to focus on talking to her on the phone.

P8

In addition to this list of strategies we discussed, clinician participants also emphasized the importance of active listening, being responsive, and spending enough time with the patient. In practice, not all clinicians can employ these strategies when they communicate with patients because of time and resource constraints, which is where empathically designed technology might be able to help fill the gap. We next turn to patients’ perspectives on what helps and does not help when they receive bad news.

### Patients’ Experience of Receiving Bad News

#### Patients’ Reactions to Bad News

When people receive a diagnosis of a severe or chronic disease, either of their own, or of their family member’s, their life changes in many ways. A patient might move to a bigger city for better care, whereas a family member might move closer to support the patient. We begin with describing a scenario of a patient who is about to learn her diagnosis. We reconstructed the scenario on the basis of P4’s experience:

A doctor walks into a room, and he is about to tell a working mother of 2 that she has Parkinson’s disease. The patient has been having trouble with small motor operations, such as unlocking a door with a key. She underwent MRI and CAT scans during a previous visit. She is waiting for the result, not knowing what the radiologist was looking for. She has been enduring a low-grade fear: fear of telling her coworkers and daughters, fear of losing her job because she is a construction inspector and her job requires driving, and fear that if she loses her job, she will also lose her health insurance coverage.

Reconstructed on the basis of P4’s experience

As portrayed in the above scenario, patient participants expressed various kinds of fears that they experienced while waiting for a concrete diagnosis of a serious disease, including fear of losing their job, losing health insurance, having to rely on others, taking regular shots, and having to use a cane, pacemaker, or feeding tube early in life. Some patient participants also experienced fear of pain, death, progression of illness, and of situations such as being chased and not being able to escape because of their condition.

Although some patients described feeling shocked at receiving an unexpected diagnosis, others, because of their perspectives from previous challenging life experiences, did the following:

...took it all in a stride

P3

Moreover, patients who visited multiple clinicians expressed *relief* at finally receiving a diagnosis that was true to their symptoms and in knowing how to manage an illness or knowing the next steps to take. For example, it took 2 years for P1 to get a concrete diagnosis of Parkinson’s disease. When she finally heard that she had Parkinson’s disease (after seeing 10 clinicians), her first reaction was a great sense of relief.

Patients also expressed feeling suddenly different. Accompanying reactions include being angry with their bodies for not working and hiding their condition and emotions from coworkers, family, and friends. P2 described the following:

I try not to show that I’m in pain, and I try not to show that I’m not feeling good because it’s just...I think it makes people feel bad to be around somebody that’s just not feeling good.

P2

In the United States, finances and the cost of care are key factors in selecting a course of treatment, especially when care is costly (eg, intensive care unit) or when procedures are not covered by insurance. Patients and family members are cautious about revealing information about health conditions in the workplace, because if they lose their jobs, they may also lose subsidized health insurance. Both patients and family members are sensitive about with whom they share the bad news. F1, a partner of P3, was an executive director of an organization. She explained why she did not want to tell her colleagues about the partner’s health situation after receiving bad news. She described it as follows:

...because of my role as executive director, every time that [Name of P3] was going through chemo, you know, I didn’t want to tell my board of directors because I thought that they would think that that would impact my performance, and it was just something that I did not want to share....I didn’t want to be seen as an absent executive director.

F1

Some patients and family members face workplace discrimination because of frequent absences and perceptions of lagging performance. Some patients can no longer work and must go on disability leave, which means adopting a new role that is different from being an employee. If patients have a severe condition, they might have to rely on others to help with shopping or driving. They might lose the freedom to walk around by themselves. When patients cannot care for themselves, they stay in a hospital. F1 eventually shared the bad news with her colleagues, and she later even called all of P3’s friends to let them know of P3’s cancer and diabetes and asked for their support. However, the initial fear and emotional fall-out that patients and family members experience at the very beginning stage of care prevent them from actively asking for and receiving the support in a timely manner.

#### Patients’ Perspectives on Poor Communication of Bad News

Patients and family participants had varying degrees of experience—in good ways and bad—in receiving bad news from clinicians. The bad experiences, in particular, were so hurtful and thus memorable that patients were able to articulate how they had felt when receiving bad news, although many years had passed since then.

Patient participants were irritated when the clinician was insensitive to their experiences and treated them like “just a number.” Patient participants eventually became angry when the clinician did not listen or asserted his/her opinion over the patients’ experience. Patient participants also expressed frustration with clinicians when the clinician did not offer sufficient opportunity to ask questions, did not answer questions, or did not adequately explain procedures, as P3 described the following:

He [doctor] definitely didn’t make some things very clear, like you know, I was kind of scared to ask him why...why aren’t you giving me these tests, why wouldn’t you give me these tests if I’d had the money.

P3

Patient participants reported that “bad doctors” are “cold,” “pompous,” and “callous” clinicians who are perceived to avoid dealing with patients or put the responsibility for communicating with patients on somebody else, disregard patients by treating them as subordinates, and prioritize clinicians’ own interests over patients’ needs. Patient participants were especially frustrated when clinicians did not spend enough time with them. P2 and P5 shared one of their experiences of receiving bad news from “bad doctors.” P2 said the following:

“I don’t have time right now,” she [the doctor] said, “talk to one of the nurses. They can answer your questions. I’m too busy...”

P2

P5 stated the following:

The very first interaction learning about it [cancer] was this very ridiculous setting which he [the doctor] was standing by the door, just to ready to leave, and saying “Oh...by the way I forgot to say, you have a cancer.” I could kill him.

P5

Poor communication of bad news left patients with more questions than answers and caused patients to withdraw and assume the issues were internal and somehow their fault. Some patient participants experienced depressive symptoms such as denial, withdrawal, and suicidal ideation. All patient participants that we interviewed, at some point in their lives, encountered clinicians who did not have a good bedside manner or empathic communication skills. When patients felt their clinician is not on their side, they sought second opinions or eventually switched clinicians. Patients also turned to other sources of comfort and built lifelong relationships with those having similar conditions, which is what we will discuss in the following section.

#### Patients and Family Members Seeking Emotional and Informational Support

After receiving a diagnosis, patients and family members sought emotional and informational support to cope with their medical condition and distress. At the time of diagnosis, it was hard for the patients and family members to know what questions to ask clinicians. In addition, clinicians often did not provide enough information, or even if they did, patients and family members are overwhelmed by the amount and content of the information, and they have a hard time assimilating it. However, as time went on, patients and family members became researchers and sought information from other sources—such as books and the internet—besides their clinicians. A patient stated the following:

And so I study a lot. I go to the library, and just look at research magazines and books that are anything related to diabetes and complications and anything like that. I get most of [it] from the internet.

P2

Another said the following:

So I’m thinking, how can I assist? I went out and, well, they’re delivering, Amazon.com, I ordered Diabetes for Dummies, and I did go with her to the meeting with the nurse and dietician...

F1

Local support groups were also a great source of information. Patients initially learned about support groups from clinicians, hospital waiting room materials, and associations’ websites. In the support group meetings, people shared an enormous amount of information that could only be learned from experienced patients who “have been diagnosed with this.” Patients talked about clinicians, procedures, drugs, and complications and obtaining information that clinicians do not give or cannot answer. Patients meet other patients from similar age groups, share their experiences, and make lifelong friendships. Patient participants described organizing special events such as children’s workshops, fundraising events, summer camps, galas, and dancing.

However, not all patient participants saw support groups so positively. A patient stated the following:

I went to the support group, but I was in denial. I mean I wasn’t accepting the fact that I was having this [Parkinson’s Disease], and I wasn’t telling anybody, and I went to a support group meeting. There were way too many people, way too overwhelming. And I didn’t like seeing the various stages of people with Parkinson’s. So I didn’t go back until October of last year...

P4

As P4 mentioned, being able to project how his/her health will deteriorate by observing other member’s conditions or being notified that a group member had deceased could make patients and family members feel depressed, uncomfortable, and prevent them from actively participating in the local patient support group.

Online health communities such as online discussion forums, live chat rooms, mailing lists, and newsgroups were also popular sources of providing emotional support and health information, which confirms existing literature [62]. However, these online health communities and online support groups imposed similar problems to local support groups in that being notified of others’ bad health conditions and their dramatic reactions could make the patient and the family member feel uncomfortable. F4 was a mother of a 4-year-old child with pediatric type 1 diabetes. She became an expert caregiver of her son, but she felt that online patient forums were not helpful for her anymore; she stated the following:

...it is not as good for me [to go to an online patient forum] because pretty much, all of those parents who just found out...they are still kind of shell-shocked...So it’s not so much as a support group. Nobody slept, everybody is shell-shocked, and everybody is freaked out...it’s kind of depressing.

F4

Information does not always equal comfort. If a patient’s diagnosis is a rare or specific one or has a grim prognosis, information from the internet and online support groups that is not specific to the patient’s situation might not be helpful, and it could even be sometimes harmful. P9 was diagnosed with Stage 4 Ewing’s Sarcoma, which is a rare type of bone cancer with a very poor prognosis. P9 stated the following:

My doctors said, “Don’t look it up. Don’t go on Internet...because it is so specific to each person. Just ask us questions directly.” And they were really good at providing me with answers. And when someone did [looked up on the internet], I took that information with a grain of salt, and said, “It’s probably not specific to me.” Because my cancer was stage 4, so it wasn’t good from the outlook from the beginning. So the Internet was not helpful.

P9

Regardless of these drawbacks, online health communities and online support groups could be a critical place for patients and family members to share personal experiences and actionable advice to cope with day-to-day health issues [63]. However, our findings about the depressing or improper use cases of patient group websites call for careful design of these sites as the information offered by other patients can only be helpful if it is accurate and tightly relevant to the inquirer’s situation.

## Discussion

### Principal Findings

Clinical guidelines for communicating health results exist to help clinicians identify strategies to help communicate bad news to patients in a way that puts patients’ emotional needs first. Clinician participants in this study tried to follow these guidelines, and when they do, they are well received by patients and their family members. Thus, there is an opportunity to apply these strategies to the design of consumer health technologies. Below, we list several design hypotheses, as recommended by Hekler et al [64], for ideas for implementing better empathic communication within technology systems that potentially communicate bad news. We call them “hypotheses” instead of recommendations or implications as they require additional testing before they can be generalizable knowledge [64].

### Design Hypotheses for Consumer Health Technologies That Communicate Concerning News

We acknowledge that not every clinical guideline can be applied in the design of health information technologies, nor do we believe that human practices can fully be facilitated by technology, but we believe technologies that may do this could be better designed. In this section, we provide a series of design hypotheses for how technologies could be designed to convey bad news and discuss how these specific design ideas can be applied to the design using health technology examples.

#### Design Hypothesis 1: Tailor the Delivery of Information to the Patients’ Individual Preferences

Patients have different information needs and personal preferences (eg, how they want to be contacted by a clinician, whether they prefer participating in online/offline support group), and clinicians can ask the patient how they would like to receive information at the time of ordering the test (eg, face-to-face, via a PHR) and when they would like to receive it (eg, as soon as it’s available, after the doctor has time to review it, etc). This aligns with the SPIKES guideline of obtaining the patients’ invitation [14].

In terms of delivering laboratory and diagnostic test results through a PHR system, we believe patients should have instantaneous access to their results, without delay, if they choose. As our patient participants stated, having a concrete diagnosis brings patients a sense of relief even though the diagnosis may be a serious disease such as Parkinson’s disease or pediatric diabetes. However, an information buffer could be placed in the system, which gives people the option to wait until a medical professional can help them accurately interpret the results with an explanation of terms (eg, the meaning of the medical terms, screening, sensitivity, and specificity) in the context of their specific health situation. For those who want to receive information verbally from a clinician, the system could send a note to the patient when the results are available and have the patient schedule a phone call or a visit. It should not be the intention to hide the information but to suggest a compassionate way of delivering a piece of potentially concerning health news by providing it at the right time. Moreover, patients need options to decide how and when they want to receive the news. A system could also provide a secure means (eg, email, voice mail) for patients to contact the clinician if they have any questions or concerns about the results and inform when the clinician will reach out to the patient. In addition, technologies could provide additional information from a trusted source where patients can begin to conduct their own research.

**Figure 1:**
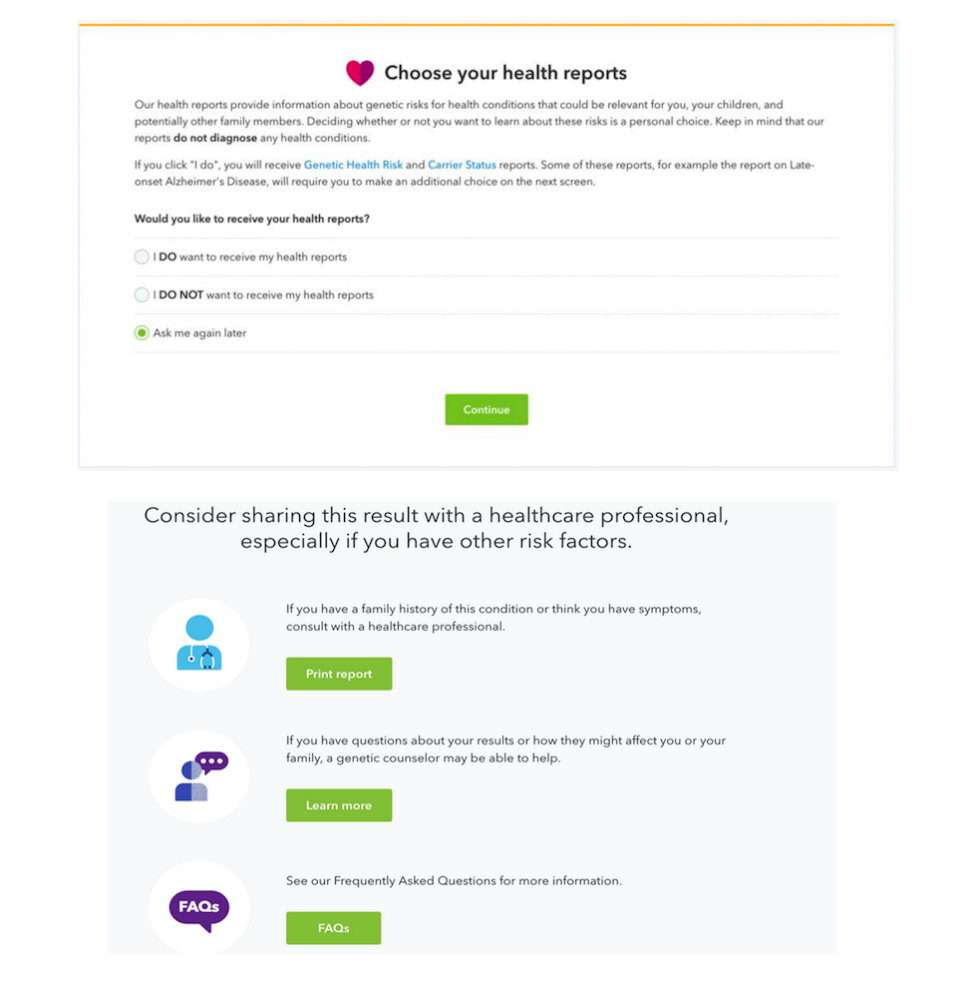
23andMe, a popular genetic testing site, adheres to several guidelines for empathic communication of potential bad news. Upper panel: the site confirms with users that they are ready to see their health results. Lower panel: 23andMe gives options for speaking with medical professionals for further information.

##### Health Technology Example

23andMe [65], a service for genetic testing, has on their website a method for delivering sensitive genetic information about increased risk of Alzheimer’s and Parkinson’s disease that aligns with this recommendation (see Figure 1, upper panel) by asking users to confirm if they would like to receive their results or not, and it explains the risks before showing the results. They also offer information on how to talk with a genetic counselor to get more information on interpreting the results (see Figure 1, lower panel).

#### Design Hypothesis 2: Support Interfaces for Tailoring Toward Patients’ Context

In this study, clinicians’ understanding of context such as patients’ feelings was an important part of empathic dialogue. Health information technologies could be designed as learning and prompting tools for clinicians to better understand patients. It was emphasized in many guidelines that it is essential for clinicians to gauge a patient’s level of understanding and emotional state during the consultation before communicating bad news. The clinicians we interviewed mentioned they were already taking notes about patients’ backgrounds and unique characteristics during medical consultations and read these notes right before the next visit. In addition, patient participants appreciated clinicians who took the time to listen to their stories and family background, which often is not necessarily reflected in the medical chart. Therefore, it could be possible to have patients add their own notes about their emotion and their background to a specific section in their medical records through a PHR. Patients could complete an electronic form where they can detail their background (eg, family history, emotional state, and preference of receiving news) in advance of the visit or while they are waiting. Future designs could tailor this over time as a patient adapts and changes preferences. This would also allow clinicians to be mindful of the patient’s emotional state or whether to invite close family members to the consultation, and it would provide an opportunity for more automated generation of tailoring news to participants’ preferences that happen remotely.

##### Health Technology Example

The field of health communication has been successful in computer-based tailoring of messages in domains such as smoking cessation [66,67], weight loss [68], and mammography screening [24] on the basis of aspects such as cultural background, gender, stage of change, marital status, whether they have children, and their social support [24,66]. As a specific example, Stretcher et al [66] found that a simple smoking cessation website tailored on the basis of baseline questionnaires participants completed at the beginning of the study was more successful than generic messages. Similarly, tailoring messaging in PHRs on the basis of user preferences and context could be used to deliver potentially bad health information in a more empathic manner by meeting patients where they are and only sharing news they are ready to hear in a manner with which they are comfortable.

#### Design Hypothesis 3: Mitigate Emotional Stress From Self-Monitoring Data

Several empathic clinicians in our study attempted to build a partnership of trust with patients and acknowledge patients’ physical and emotional discomfort. However, when people receive personal health information from commercial self-monitoring tools, they do not have a counterpart of a care provider who can provide emotional and informational support. While using self-monitoring tools, people might feel distressed when they find they have not met their weekly goals or when they feel what they have been experiencing is abnormal. Take an example of a patient who is experiencing severe pain after surgery and is monitoring his pain level. Feedback from a pain tracking system could convey information about what is normal in plain language (eg, “80% of people experience severe pain after this surgery”) with an aim to lower the patient’s distress. Interfaces could also use language that reinforces an “us” relationship similar to what our clinician participants stated. For example, when a glucometer presents a higher than normal blood glucose reading, the interface could say, “A single high blood sugar reading usually isn’t a cause for alarm, but let’s check a few things together,” and guide the patient through possible reasons—medication, food, and exercise.

##### Health Technology Example

**Figure 2:**
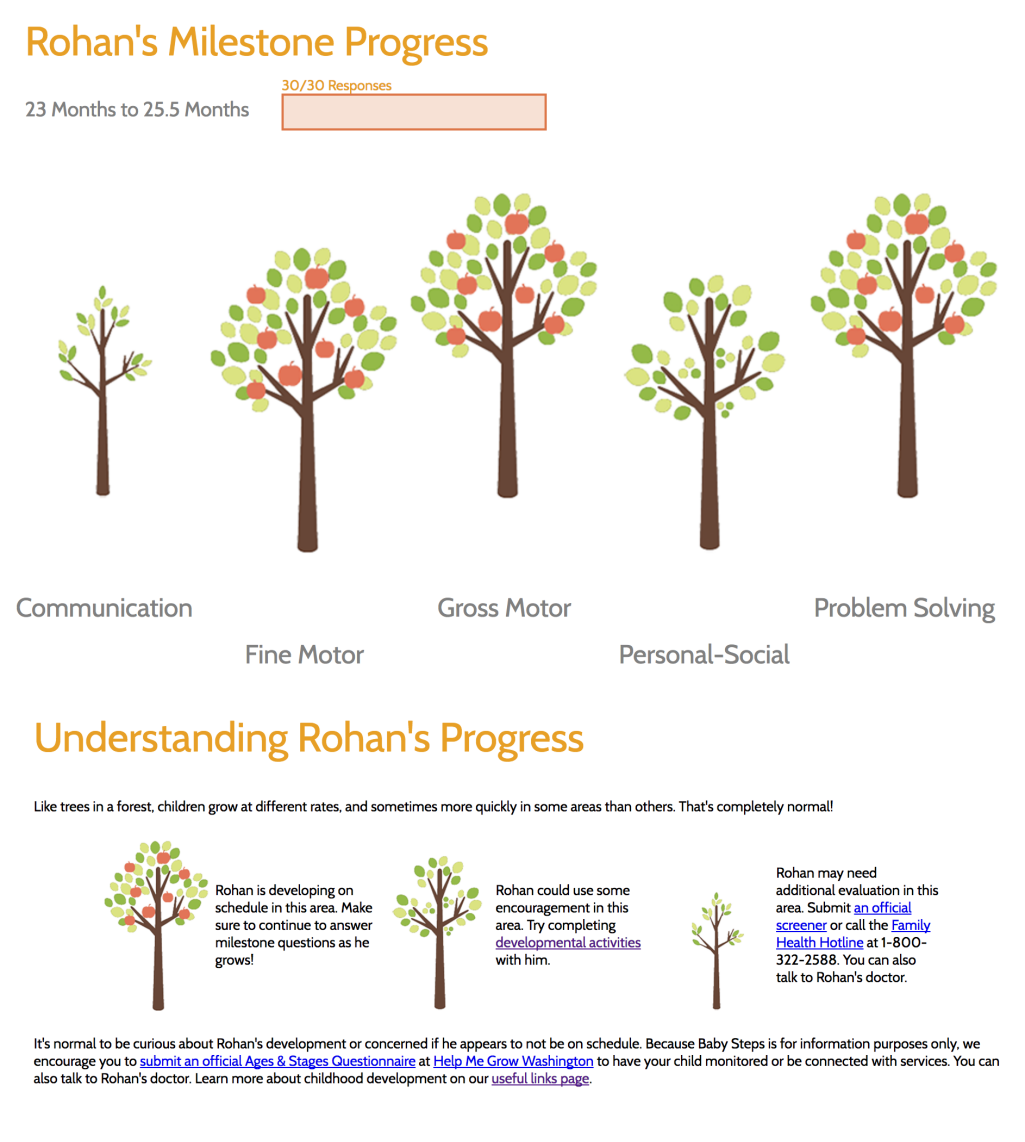
Interface for conveying the results from a developmental screen in the Baby Steps Web portal. The different sized trees represent where a child is at developmentally for a given category. Immediately below the trees is an interpretation that uses team-based language, acknowledges the potential for anxiety, and indicates that variation is normal development.

On the developmental screening results page for Baby Steps [69,70], we use language that acknowledges that it is normal to feel anxious about how your child is doing developmentally and provide some sense of what is normal, which might cause potential for worry but is not actually worrisome (eg, variation across categories, small plateaus, not answering “yes” to all screening questions). We also use “we” language to emphasize a partnership in tracking children’s progress and working together to accomplish the task of monitoring children’s development. For example, language describing how to interpret the visualization of the results states, “Rohan could use some encouragement in this area. Let’s find some developmental activities to try with him.” We also tested early screen mockups of different visualizations of the results for developmental screening with parents in a Web-based survey. The resulting visualization that received a high level of understanding of the results and also reduced anxiety was a more abstract visual metaphor to communicate the child’s developmental progress where different sizes of trees represent the child’s growth (see Figure 2). This visualization used the metaphor that children grow at different rates, and a lower score on a developmental screen may just mean that their child has not yet had the opportunity to grow in a given area. Currently, as there is no evidence on the fact that hitting milestones earlier has an impact later in life [71,72], we chose to only communicate results if a screen indicated children were at risk of developmental delay and needed further evaluation or needed to be encouraged with developmental activities rather than showing exact percentiles.

#### Design Hypothesis 4: Help Identify Clear, Actionable Steps Patients Can Take Next

Some patients in this study reported feeling helpless when they received bad health news that was communicated poorly and that they expressed a desire for things they could do to feel less helpless. Moreover, 1 way to accomplish this would be to help patients by giving them clear, actionable steps they can take after receiving a diagnosis. This could be as simple as giving them trusted information they can read more about, suggestions for contacting a close family member or counselor and instructions for what to say to get support, or actions they can do to start treatment, such as scheduling an appointment with a clinician.

##### Health Technology Examples

Overall, 2 of the previous technologies we described have good examples of this design recommendation in practice. For 23andMe [65], patients are given the option to talk with a genetic counselor directly through the site on the basis of the results of a genetic screen (Figure 1, lower panel). With Baby Steps (Figure 2, bottom), we couple results from a developmental screen with information for the parents immediately on the screen where they see the result. If the result that the child is close to the cut off for having a developmental delay, Baby Steps links parents to a list of activities they can do with their child that encourage development, which they can check off as they complete them. If the result is that they need an evaluation beyond self-monitoring, parents are linked to free services they can contact, which will help them to conduct a more formal evaluation, and they are given the number to a toll-free parent help hotline they can use to talk to someone immediately.

### Conclusions

The objective of our research was to uncover insights for the design of health technologies that potentially convey concerning news. We accomplished this goal by (1) examining established guidelines for clinicians on communicating bad news related to health, (2) conducting interviews with patients, patients’ family members, and clinicians on their experience of delivering and receiving a diagnosis of a serious disease, and (3) rethinking the design of health information technologies—EMRs, PHRs, and self-monitoring tools—to support clinician-patient empathic dialogue and reduce the discomfort of patients when they receive bad news. We have addressed how the human element is conveyed during medical practice, especially when communicating diagnoses of severe or chronic diseases. We also identified how clinicians develop their own strategies to understand patients and communicate with them, and we investigated patients’ internal turmoil and emotional distress when receiving bad news and emotional and informational support that patients and family members seek elsewhere. We tied our findings to 4 design hypotheses for health technologies aimed to facilitate better self-managed care and promote the expression of empathy in the clinical setting, and we demonstrated their application in different health technology designs. We believe that future work might be to explore these design hypotheses and validate both positive and negative technology examples empirically with potential users as well as explore how strategies for empathic communication might evolve over time.

Empathic communication should be considered a core value in the design of health technologies [73], and a more empathic approach to design is needed [74]. Patients’ needs and their situations are different and a “one-size-fits-all approach” does not work. However, health information technology has a great potential to support and reinforce the empathic relationship of a clinician and patient. Our approach of investigating the best-case practices of empathic communication is the first step to bringing “empathy” into the designs of empathic health information technologies.

## Abbreviations

EMRs: electronic medical records
PHRs: personal health records
